# Thoracentesis under clopidogrel is not associated with excessive bleeding events: a cohort study

**DOI:** 10.1186/s12931-020-01549-z

**Published:** 2020-10-27

**Authors:** Sivan Perl, Marina Bondarenco, Noam Natif, Yitschak Shpirer, Sharon Enghelberg, Benjamin Fox

**Affiliations:** 1 Pulmonary Institute, Shamir Medical Center, 70300 Tzrifin, Israel; 2 Sackler Faculty of Medicine, Tel Aviv University, Tel Aviv, Israel; 3 Internal Ward C Shamir Medical Center, Tzrifin, Israel

**Keywords:** Thoracentesis, Clopidogrel, Bleeding complications

## Abstract

**Background:**

Thoracentesis is a low-risk procedure for bleeding (approx. 2%). Data regarding safety of thoracentesis under treatment with clopidogrel is scarce, and current guidelines are not evidence based. We performed a retrospective study to evaluate the rate of bleeding complications of thoracentesis under clopidogrel in hospitalized patients.

**Methods:**

Retrospective chart review of hospitalized patients undergoing thoracentesis with or without clopidogrel treatment. Demographic and clinical data, diagnostic ICD9 codes, and use of ultrasound were extracted. Bleeding endpoints were defined as hemothorax, drop of > 2 g/dL hemoglobin, or need for packed red cell transfusion.

**Results:**

The study group comprised of 88 cases and 169 controls. Four bleeding complications were noted in the cases group, versus 5 in the control group (RR 1.53, 95% CI 0.4–5.5).

**Conclusion:**

Thoracentesis may be performed safely in patients receiving clopidogrel. Bleeding event rates are consistent with previous reports of thoracentesis in general.

## Introduction

Thoracentesis is a very commonly performed diagnostic and therapeutic procedure, with a low risk of complications (0–39% in older studies [1], up to 2% in recent studies [2]). The rate of bleeding complications ranges between 0.5 and 3% [1, 2]. Clopidogrel is a thienopyridine anti-platelet agent indicated for a wide range of vascular diseases. It is therefore a common occurrence that a patient requiring thoracentesis will be treated with clopidogrel. Withholding clopidogrel for an invasive procedure may confer an increased risk for a recurrent vascular event [3]. Current guidelines recommend withholding clopidogrel treatment for 5 days before performing thoracentesis [4], but these recommendations are based on low quality evidence. There are few, mostly uncontrolled studies, with small numbers of patients and no control group, all of them suggesting that there is no excessive risk of bleeding performing the procedure under clopidogrel [5–10].

Our study goal was to estimate the bleeding risk of thoracentesis under clopidogrel in a large group of patients in comparison with a control group.

## Methods

We identified potential hospitalized patients by querying the laboratory database for samples of pleural fluid arriving at the hospital laboratory between 1/1/2015 and 31/7/2017. For all subjects we extracted demographic data (age and sex), concomitant medication of anti-platelet or anti-coagulant agents used at the time of thoracentesis and use of ultrasound during the procedure. Charlson comorbidity index was calculated for each subject [11, 12]. All thoracentesis procedures performed under clopidogrel treatment were allocated to the cases group, and all those performed without clopidogrel were allocated to the control group. The primary endpoints were bleeding complications, prospectively defined as hemothorax requiring drainage, hemoglobin drop of > 2 g/dL, or need for packed red cell transfusion. Secondary endpoints were complications not related to bleeding, e.g. pneumothorax, etc. We used these endpoints as surrogate markers of safety of thoracentesis. We used the fragility index to evaluate the robustness of the findings [13].

Statistical analysis was performed using the R statistical system (v 3.3.4) with the meta and fragility index packages [13, 14]. Fisher’s exact test was used to compare categorical data between case and control groups. Relative risks were calculated. Wilcoxon rank sum test was used to compare continuous variables of both groups. p value of < 0.05 was considered significant.

The study was approved by the hospital’s local ethics committee.

## Results

Eighty eight cases of thoracentesis under clopidogrel treatment were identified during the study period and included in the case group. One hundred and sixty nine cases of thoracentesis without clopidogrel treatment were identified and included in the control group. Mean age of the study group was 77.5 years (SD ± 11), and 76 years in the control group (SD ± 14) (p = 0.33). Patients in the two groups were similar except for slightly higher Charlson comorbidity score (p < 0.001).

Regarding the use of other medications affecting coagulation, there was higher exposure to aspirin in the cases group (p < 0.001) (see Table 1). Few patients were on DOAC therapy—in all cases the drug was held for 24–48 h before the procedure. Few patients were on warfarin treatment prior to the procedure, two of them had INR of 1.5 or less during the thoracentesis, one received fresh frozen plasma (FFP) before the procedure, and two had the procedure done with no INR data or drug cessation (one in each group).

**Table 1:** Demographic and clinical data of study population

	Clopidogrel treatment (n = 88)	No clopidogrel treatment (n = 169)	p value
Age (mean ± SD)	77.5 ± 11	76 ± 14	0.33
Sex (male)	53 (60%)	82 (48%)	0.09
Charlson comorbidity score (median, IQR)	2 (1–3)	1 (0–2)	< 0.001
Concomitant drug use			
Aspirin	41 (46%)	9 (5%)	< 0.001
Warfarin	2 (2.3%)	3 (1.7%)	1
DOAC	2 (2.3%)	4 (2.4%)	1
Other bleeding risk factors			
Platelet count (mean ± SD)	249 ± 124	259 ± 129	0.72
Renal failure (n)	26	15	< 0.001
US guidance	22 (25%)	57 (34%)	0.15

*IQR* inter quartile range, *DOAC* direct oral anti coagulants, *US* ultrasound

Regarding other bleeding risk factors, thrombocyte count was similar in both groups (see Table 1), with minimum count of 63 K cells in both groups. There was higher prevalence of renal failure in the cases group (p < 0.001) (see Table 1). One patient (in the control group, not on aspirin or any other anti-coagulant) received 6 units of platelets before the procedure due platelet count of 66 K.

### Primary endpoint

The total bleeding event rate was similar in both groups (p = 0.49, RR 1.53, 95% CI 0.4–5.5). 2.2% of patients in the cases group required packed cells after the procedure, vs 1.2% in the control group (RR = 1.9, 95% CI 0.3–13.4, p = 0.6). 2.2% of patients in the study group developed a drop of hemoglobin level of > 2 g/dL, vs 1.2% in the control group (RR = 1.9, 95% CI 0.3–13.4, p = 0.6). None of the patients in the study group developed hemothorax following the procedure, vs 1 patient (0.6%) in the control group (RR = 0.63, 95% CI 0.02–15.8, p = 1). Rate of bleeding events was not dependent on use of ultrasound guidance during the procedure (6% of procedures under ultrasound, versus 2% without (RR = 2.9, 95% CI 0.8–10.4, p = 0.13) (Table 2).

**Table 2:** Bleeding events and total outcomes of thoracentesis under clopidogrel versus controls

	Clopidogrel treatment (n = 88)	No clopidogrel treatment (n = 169)	RR (95% CI)
Bleeding endpoints			
Required packed cells	2	2	1.9 (0.3–13.4)
Hemothorax	0	1	0.63 (0.02–15.8)
> 2 g/dL drop in hemoglobin	2	2	1.9 (0.3–13.4)
Total bleeding events	4	5	1.53 (0.4–5.5)
Other complications, not bleeding related			
Pneumothorax	3	2	2.9 (0.5–17)
Re-expansion pulmonary edema	1	0	5.7 (0.2–139)
Total complications	7	7	1.9 (0.7–5.3)

We performed an unplanned post hoc analysis for the interaction of aspirin use and bleeding risk due to the significant difference of its use in both groups. There was no interaction found between aspirin use and the risk of bleeding in both groups (p = 0.7 in clopidogrel group, p = 1 in control group).

### Secondary endpoints

Other complications noted were pneumothorax and re-expansion pulmonary edema. Rate of pneumothorax was 3.4% in the study group vs 1.2% in the control group (RR = 2.9, 95% CI 0.5–17, p = 0.34). Re-expansion pulmonary edema developed in one case in the cases group vs none in the control group (RR = 5.7, 95% CI 0.2–139, p = 0.34).

In a post hoc analysis we used the reversed fragility index to calculate the number of cases of any bleeding end point needed to make a non-significant result significant. The reverse fragility index is 4, implicating that the number of bleeding cases needs to be doubled in order to change the results of the study, hence enhancing the strength of our findings.

## Discussion

We performed a retrospective cohort study to compare bleeding complications in clopidogrel treated patients undergoing thoracentesis. We found that thoracentesis under clopidogrel treatment was not associated with higher rate of bleeding complications when compared with controls. This finding is important because there is very little data to inform guidelines. The BTS guidelines on pleural procedures are silent regarding this issue [15]. General guidelines for periprocedural management of coagulation status and hemostasis risk in percutaneous image-guided non-vascular interventions (such as thoracentesis), recommend withholding clopidogrel for 5 days before the procedure [4]. Most of references quoted in this guideline are not specific for thoracentesis. Our data in a large cohort suggest that these guidelines may be overly restrictive since clopidogrel exposure did not significantly increase bleeding. Since withholding clopidogrel may increase risk for vascular events and confer a risk for the patient, this recommendation could, in principle, paradoxically worsen the overall outcome and prognosis.

Other previously published studies regarding thoracentesis under clopidogrel found a low rate of bleeding complications [5–10]. However, these studies were small, including up to few dozens of patients (n = 7–69), and most of them did not include a control group. Our study represents the largest report of patients who had the procedure done under clopidogrel versus a control group, with the same low bleeding complication rate. Having said that, it should be noted that the absence of difference between the groups should be interpreted cautiously due to the small number of events in both groups, possibly resulting in of lack of power.

Moreover, 46% of patients in the study group were receiving aspirin as well as clopidogrel at the time of the procedure (versus only 5% in the control group, p < 0.001). The higher prevalence of aspirin use in the clopidogrel group is well understood since patients under clopidogrel treatment usually have vasculopathy, and it is reasonable to expect high use of aspirin in this group. The risk of bleeding was still similar in both groups even though almost half of the patients were under dual anti platelet therapy. Since the difference in aspirin use might confer a possible confounding factor, we performed a post hoc analysis to rule out a possible interaction between the use of aspirin and risk of bleeding. There was no interaction found between aspirin use and the risk of bleeding in both groups. These data strengthen our findings, that anti-platelet therapy in general may not be regarded as a contraindication for thoracentesis.

We noted significant differences between the study groups in the Charlson comorbidity index score, which was higher in the study group (median, 2 in study group versus 1 in control group, p < 0.001). This difference is easily explained by the fact that cardiovascular diseases (myocardial infarction, chronic heart failure and cerebrovascular event/transient ischemic attack) are all included in the Charlson comorbidity index score and would be likely to be treated with clopidogrel. Intuitively, patients that are sicker would be expected to have higher tendency for complications.

For example, renal failure, as another bleeding risk factor, was more prevalent in the cases group, as reflected in higher Charlson scores. Even though renal failure was more prevalent in this group, the bleeding events was similar between groups, suggesting that chronic renal failure is not an independent risk factor for bleeding in these patients.

It should be noted that the use of US was limited in this cohort. Even though the use of US to guide thoracentesis is considered to be a state of the art practice these days, the data is taken from the practice of medicine wards during 2015–2017, while the instrument was not generally available in our center, hence the low rate of use. Nevertheless, the rate of bleeding complications was not different when the procedure was done with or without US guidance.

This study has some limitations. First, it is a retrospective, single center study. However, it would be ethically and practically challenging to design a prospective study to answer this question, even though it may produce more solid evidence. Such a study design may be more feasible now, based on our safety findings. Second, we included only hospitalized patients, which may affect the ability to generalize the conclusion to ambulatory patients. As suggested above, sicker (hospitalized) patients would be expected to have a higher bleeding risk than ambulatory patients thus we do not think that this would affect our conclusions. Third, the needle bore used for the procedure is not standardized, and different needle sizes may have been used in the procedure, a factor that may influence bleeding risk. The range of needle bore, however, was not large, ranging from 18 to 23G (most cases 18G–21G), and would probably have been decided according to local practice rather than due to clopidogrel treatment. Again, we do not consider that needle bore would be a confounding factor since the choice of needle was by departmental preference and not according to concomitant medication.

## Conclusion

In this cohort study, thoracentesis under clopidogrel treatment had a low rate of bleeding complications, which was similar to controls. Since stopping clopidogrel may be harmful for the patients, this data is of special importance. Even dual anti platelet treatment seems to be safe, even though this study was not designed to answer this question directly.

We suggest, that in the absence of randomized clinical trial data, it may not be necessary to stop clopidogrel treatment before thoracentesis.

## Data Availability

The datasets used and/or analysed during the current study are available from the corresponding author on reasonable request.
